# Non‐European immigrants' self‐described strategies for mental health promotion and perceptions of Finnish mental health services: A qualitative descriptive analysis

**DOI:** 10.1111/ppc.13096

**Published:** 2022-04-21

**Authors:** Minni Roth, Mari Lahti, Noora Gustafsson, Johanna Berg, Elina Kaitala, Lauri Kuosmanen

**Affiliations:** ^1^ Department of Nursing Science, Faculty of Health Sciences University of Eastern Finland Kuopio Finland; ^2^ Department of Nursing Science University of Turku Turku Finland; ^3^ Department of Nursing Turku University of Applied Sciences Turku Finland

**Keywords:** Finland, immigrant, mental health, mental health services, nursing

## Abstract

**Purpose:**

This study aims to describe non‐European immigrants' perceptions toward mental health care in Finland and the factors that support their mental health.

**Design and Methods:**

Participants (*N* = 17) were interviewed through semistructured interviews, after which interview transcripts were analyzed using a six‐phase thematic analysis process.

**Findings:**

The participants reported that developing self‐help strategies benefitted their mental health. The participants primarily preferred unprofessional help over professional mental health care. Several structural and cultural barriers to help‐seeking were identified.

**Practical Implication:**

Health care staff working with immigrants need proper education and knowledge to provide sufficient, holistic care; for this reason, information about the cultural differences among immigrants and cultural sensitivity should be included in basic as well as post‐graduate nursing education.

## 1 INTRODUCTION

Immigrants, that is, people who voluntarily or involuntarily change their country of usual residence (United Nations [UN], 2019), may undergo a stressful immigration process that predisposes them to several mental health risks (World Health Organization [WHO], 2018a). As a result, a higher prevalence of mental health problems has been observed in immigrants relative to the general population (e.g., Pampati et al., 2018; Von Werthern et al., 2018). For instance, research covering six European countries reported that immigrants are 2.5 times more likely to develop mental disorders than native populations (European Observatory for Health Systems and Policies, 2011).

Immigrants are a heterogeneous population that includes economic immigrants, refugees, and asylum seekers, among others (the International Organization for Migration [IOM], 2020). Despite a well‐documented need for mental health services, immigrants often use these services less than the general population (e.g., Derr, 2016; Kieseppä et al., 2020), while numerous barriers—for example, language, lack of information, attitudes, financial difficulties, logistical issues, lack of knowledge on how the local health system works, long waiting lists, dissonance between the cultural systems of the country of origin and the host country, and stigma—may prevent immigrants from accessing mental health services (Kiselev et al., 2020; WHO Europe, 2018a). Both of these factors result in unmet mental health care needs among immigrants (Lebano et al., 2020).

International immigration has increased during the last decades (WHO, 2019). Nearly 920 million people live in the WHO European Region, with approximately 10% representing immigrants (WHO Europe, 2018b). The need for mental health services among immigrants has increased linearly with the growing immigration rates. Good mental health is associated with quality of life (Van Der Boor et al., 2020) and is also pivotal for immigrants' successful integration (European Observatory for Health Systems and Policies, 2011). The successful integration of immigrants is an important issue for all European states, with the legal, moral, and economic participation of immigrants crucial to the future well‐being, prosperity, and cohesion of European societies (European Commission, 2016).

## 2 BACKGROUND

Research on mental health care service utilization by immigrants has mainly covered the barriers to treatment, with only a few studies examining which factors facilitate access to care (Byrow et al., 2020). The barriers to mental health and psychosocial support services reported by immigrants include a lack of information, language difficulties (e.g., Doğan et al., 2019; Kiselev et al., 2020; Satinsky et al., 2019; Valibhoy et al., 2017), and negative attitudes, which can also include care providers' attitudes (Doğan et al., 2019; Satinsky et al., 2019). In addition, a lack of cultural competency among health care professionals and financial costs (Kiselev et al., 2020; McCann et al., 2016) have been reported to impede access to mental health services. Concerning care givers, their perceptions of immigrants (Bartolomei et al., 2016) and a lack of knowledge about mental health problems among immigrants (Kiselev et al., 2020) may reduce immigrants’ access to mental health care.

Immigrants have also reported that the health care system is not always aligned with their perceived needs (Aggarwal et al., 2016; Kiselev et al., 2020). For example, certain problems—such as depression or anxiety—may not be viewed as an illness (Kiselev et al., 2020). Thus, immigrants often prefer other options (Satinsky et al., 2019; Valibhoy et al., 2017), such as religious healing (Bettman et al., 2015; Mölsä et al., 2017) and/or psychosocial support from their own network (Renner et al., 2020), instead of using official health services.

Besides barriers, certain factors are conducive to help‐seeking among immigrants with mental health problems, namely, being open with friends and family that can facilitate help‐seeking among immigrants (McCann et al., 2016). Furthermore, increasing mental health literacy, ensuring confidentiality, and engaging the family and community in care, might improve the rates at which immigrants seek help for mental health problems (Colucci et al., 2015; McCann et al., 2016). Decision‐makers should improve accessibility to care (Colucci et al., 2015; Valibhoy et al., 2017) and provide interpreters (Colucci et al., 2015) with adequate training (Wamwayi et al., 2019) if health care systems are expected to respond to the unmet needs for mental health services among immigrants.

Future mental health services should be culturally appropriate (Mölsä et al., 2017), while immigrant population will require specialized mental health care services (Kien et al., 2019). In general, this means that health care systems need to become more flexible and should embrace innovative development paths to serve diverse target populations (Kiselev et al., 2020). Moreover, service development should engage the target population as it would be erroneous to assume the treatment preferences of patients without asking for their input (Aggarwal et al., 2016).

In 2019, approximately 8% of Finnish residents had a foreign background (Statistics Finland, 2020). Furthermore, previous reports indicate that—in Finland—the prevalence of mental health problems is higher among the immigrant population than the general population, yet immigrants utilize mental health services less than other members of society (Finnish Institute on Health and Welfare, 2020). Thereby, this study aims to describe non‐European immigrants’ perceptions of Finnish mental health care and the factors that support their mental health. The ultimate goal of the presented research is to provide knowledge that can be used to support the mental health of the immigrant population in Finland.

The questions underlying the research are as follows:

(1). Which factors promote mental health among the non‐European immigrant population in Finland?
(2). How do non‐European immigrants in Finland view mental health services?

## 3 DESIGN AND METHODS

### 3.1 Study design

The presented research applied a descriptive qualitative approach (Polit & Beck, 2012), which is commonly used when gathering information on participants' experiences, opinions, and perceptions of the studied phenomenon (Palinkas, 2014; Polit & Beck, 2012).

### 3.2 Sampling, study population, and procedure

The participants were recruited via several third sector nongovernmental organizations working with immigrants in southern Finland. Recruitment notifications, including contact information, were placed in the bulletin boards of the organizations. Researchers also visited the organizations to host events during which potential participants could ask questions and volunteer to participate. In addition, staff at these organizations disseminated information about the study and, thereby, were involved in the recruitment process. Participants could sign up to participate in this study either through the researchers or staff working at the organizations, who would then contact the researchers and schedule an interview time.

Purposive sampling (Polit & Beck, 2012) was used to recruit participants representing a wide range of immigrant backgrounds, as immigrants are not a homogeneous population. Participants were then selected based on their ability to provide the requested information. The inclusion criteria for participation were: (1) at least 18 years of age; (2) voluntarily participating in the study; (3) the participant, or both of his/her parents, was born in another country not included in the European Union; (4) being able to participate and give informed consent; and (5) being able to understand and speak Finnish, Swedish, or English.

A total of 17 participants took part in this study. Out of these 17 participants, 1 was male and 16 were females. The average age of participants was 34.8 years, which varied between 23 and 58. The participants had varying educational levels, ranging from no education at all (*n* = 3), secondary school (*n* = 2), high school (*n* = 2), adult education (*n* = 4), vocational education (*n* = 2), to a bachelor's degree (*n* = 2), and other (*n* = 2). They had arrived in Finland between the years 1989 and 2017, and had spent an average of 13 years in the country. One of the participants was born in Finland to parents who had immigrated from an non‐European country. The participants were originally from Afghanistan (*n* = 1), Guinea (*n* = 1), Iran (*n* = 1), Iraqi Kurdistan (*n* = 2), Japan (*n* = 1), Kosovo (*n* = 1), Pakistan (*n* = 1), Somalia (*n* = 8), and Vietnam (*n* = 1).

### 3.3 Data collection

Data were collected between May and July 2019 using semistructured individual interviews (Polit & Beck, 2012) (*n* = 13) and joint interviews (Morgan et al., 2013) (*n* = 4). The interviews covered the following themes: (1) perceptions of the factors that support mental health; (2) treatment of mental health problems; and (3) things to consider when organizing mental health services for the immigrant population. Interviews were conducted either at the participating organizations or in the premises of Turku University of Applied Sciences. The researchers (E. K., J. B., and M. R.) conducted the interviews according to a joint protocol to ensure a similar setting across the different interviews. For example, the interviews were conducted in a peaceful environment and the questions were always asked in a similar way. If necessary, the questions were repeated to ensure mutual understanding, and the participants had a chance to ask about the meaning of the question if they did not understand it. The interviews lasted from 13 to 51 min. All of the interviews were recorded and transcribed. Most of the interviews were conducted in Finnish, with one conducted in English.

### 3.4 Data analysis

The collected data were analyzed using a six‐phase thematic analysis process presented by Brown and Clark (2006). First, the researchers familiarized themselves with the data, that is, the written transcripts from the interviews were read through several times to obtain a general understanding of the material and formulate tentative ideas of the content. During Phase 2, the researchers produced initial codes from the data while keeping the research questions in mind, for example, which factors support mental health and views of professional mental health care in Finland. In the Phase 3, the researchers analyzed the codes and organized them into categories by clustering related codes into broader themes. Next, in Phase 4, the researchers reviewed the themes, first based on how they were related to the coded data and secondly based on how they were related to the entire data set, to ensure they form a coherent patter. During the Phase 5, the themes were defined and named, while Phase 6 entailed the reporting of the results (Brown & Clark, 2006). An example of the thematic analysis process is presented in Table 1.

**Table 1:** Example of the thematic analysis process

Original line	Code	Category	Main theme
*“…*I take a walk in the forest*.”*	Going out	Meaningful activities	Promoting mental health
*“…*I don't know where to go*.”*	Lack of knowledge	Structural barriers	Barriers to help‐seeking

## 4 ETHICAL CONSIDERATIONS

Good scientific practices and principles of publication ethics were followed throughout the research process (ALLEA, 2017). Ethical approval was obtained from the ethical committee of Turku University of Applied Sciences (2/2019). Participants received both written and verbal information before they were asked to participate. Participants volunteered to take part in this study and signed a form of informed consent regarding data collection via interviews and taping of the interviews. The participants were also allowed to drop out from the study at any point. The researchers highlighted participants to respect the privacy of fellow participants and the data were reported so that participants could not be identified. The researchers obtained permission to conduct the study in a way that was aligned with organizational policies.

## 5 RESULTS

The analysis identified four themes from the data, namely, (1) promoting mental health, (2) preferring unprofessional help over professional services, (3) barriers to help‐seeking, and (4) hopes and ideas for service development.

### 5.1 Promoting mental health

Participants developed self‐help strategies that they considered beneficial to their mental health. These included simple, yet meaningful, activities, positive thinking as well as engagements with family and friends. Many of the participants' comments featured how spending time in nature is beneficial to their mental health and helps manage stressful situations. The participants gave various examples of their experiences in nature, for example, going out for walks, taking a trip to the forest, and going to the seaside “If I take a walk in the forest” (P14).

Besides nature, the participants mentioned different activities, such as crafts, having a hobby, participating in activities arranged by third sector organizations, exercising, cooking and baking, playing with kids, and listening to the Quran, that promoted better mental health. The participants mentioned that they would engage in these activities when feeling sad, depressed, or anxious.

This place (third sector organization) is good for you, I always tell my friend how it is really good here. (P1)

In addition to being active, the participants mentioned how taking time for yourself was equally important and helped them cope with stress.

For me it is very important that I find time for myself… even if it is just a couple of hours to relax. (P17)

Furthermore, trying to focus on positive things was seen as essential to good mental health. The participants stressed that it is important to focus on all the positive things in life instead of the negative things, and to be satisfied with what you have, no matter how little it might be.

So I try to be positive until bad things happens. People tend to feel negative or depressed before something happened, just thinking about it. I think it's kind of wasting time, because if it didn't happen, it is a waste of time to think about it. (P16)

The participants also stressed that not worrying about what might happen in the future is essential to mental health. A few of the participants suggested that worrying may actually cause some mental health problems.

Have to think about good things, and not always sorrow or bad things … because you get a bad mood every time you think about illness or bad things. (P1)

The participants also highlighted the importance of their social community. For example, participants considered engagements with family and friends crucial to good mental health, with almost all of the participants mentioning this aspect. During these engagements, people would gather around to socialize and communicate with one another, thus providing support and comfort to those in need, along with practical help such as cleaning or cooking. Family members, friends, and loved ones create a big community in which no one feels alone, and help is provided whenever it is needed. Furthermore, any possible problems that might occur would be discussed with family and friends. Correspondingly, the participants noted that the loneliness and isolation caused by being separated from their families is highly stressful and causes anxiety.

But there is always family, always the aunt, father, wife, uncle, everyone, yes. (P2)

And when I see my friends or mum, my sister, and when I talk to someone, it gives me a good mood. (P9).

### 5.2 Preferring unprofessional help over professional services

In relation to treatment for mental health problems, the participants of this study primarily preferred unprofessional mental health care, that is, that provided by themselves, family members or religion, over professional help. The participants felt that they were capable of treating their own mental health problems, with some expressing that it was their own responsibility, or a family responsibility, to care for mental health problems. Few participants reported having been offered professional mental help, but they all declined because it was considered unnecessary. “Where do I get help? from a doctor or where? If it is just a little depression, I would not seek help” (P9).

Professional help was seen as a grave, almost radical option, with a majority of the mental health problems considered so mild that neither medical nor professional care would be necessary. In contrast, self‐care, care from the family or religious help were perceived as sufficient care for most situations or should at least be tried out first. Despite these perceptions, many of the participants were familiar with situations in which professional help had resulted in positive outcomes. “At first Quran, this person is ill” (P6).

A majority of the participants knew that help could be requested from a doctor, psychiatric nurse, or psychologist at health care center. However, this approach was seen as the last resort. The participants reported that all possible self‐help strategies should be tried before going to doctor could be considered.

But if it is really bad then the doctor is needed. (P2)

### 5.3 Barriers to help‐seeking

A majority of the participants stated that there is no reason why they would not seek professional help for their mental health problems if they could not solve the problem by themselves. However, some structural and cultural barriers to help‐seeking were identified through the thematic analysis, including a lack of knowledge regarding services or mental health problems and language difficulties.

If I'm in the situation then I don't know where to go. (P18)

Furthermore, the participants acknowledged possible prejudices against the native population and stigma associated with being an immigrant. “When people are afraid of that shame and stigmatization” (P15).

### 5.4 Hopes and ideas for service development

The participants hoped that the mental health services would be more flexible and tailored to immigrant needs. As possible prejudices against native Finns were described, the results of the thematic analysis suggest that mental health services should also recruit staff members from the immigrant population. In addition, the immigrants would like be able to access mental health care services in their native language, as talking via a translator was considered uncomfortable. On the other hand, translators have an important role in integrating immigrants into society; hence, they could also be educated in mental health issues and subsequently utilized to guide immigrants on mental health issues.

So maybe hypothetically, if a group would be organised, then we would be able to communicate in the same language. (P15)

In addition, services aimed at different generations of immigrants could potentially better serve the care needs of immigrants, as young immigrants could be more open‐minded on mental health issues compared to older immigrants. Moreover, older immigrants might benefit from therapeutic group sessions, as one‐on‐one discussions might be experienced as awkward. Also, participating in a group session could afford immigrants peer support when discussing difficult issues, such as traumatic war experiences.

Maybe if you come from a war area, you have traumatic experiences, then you can share them. If it is considered oppressive, then the one‐on‐one session can become awkward. If you would go through these experiences in a group then everyone can share these vibes. Then it is easier to acknowledge that you are not alone in these things. (P15)

Participant descriptions also featured a need for versatile information. In addition to general information, the results of the thematic analysis revealed that immigrants want information concerning the difference between mental health and religion, women's rights, and the ability to make one's own choices. Meeting this demand would require that health care staff have sufficient knowledge of these topics

somehow probably to highlight the difference between religion and mental health. (P8)

In addition, health care staff need to be educated in recognizing mental health care issues among immigrants.

It is known that within the last years extremely traumatic events have occurred on the other side of the continent [in the middle East]. And the evaluation of these care needs requires, you know, skilled workers. (P16)

The results of the thematic analysis also suggest that mental health services should have a low threshold for receiving help. For example, these services could be integrated with different social and immigration services to encourage more people to receive professional help.

So that you would start with some preventive low‐threshold service, that would not necessarily, maybe through some activity, show that it is a mental health service. (P8)

## 6 DISCUSSION

The findings of the present study shed light on immigrants' perspectives of which factors they consider to support their mental health. Mental health promotion and understanding which factors positively influence mental well‐being are both essential to preventing mental health problems (WHO, 2013). While immigrants’ mental health can be promoted on a societal level through, for example, political decision‐making concerning social integration (WHO Europe, 2018a), small, everyday actions were also identified to relieve stress. It is noteworthy that despite cultural differences, some of the factors that support mental health identified by participants, such as enjoyable activities, align with previous findings from different populations (Fusar‐Poli et al., 2020; Shimazaki et al., 2020). It has also previously been argued that nature can positively impact mental health by relieving stress, aggression, and mood‐related problems (e.g., Bratman et al., 2012).

Furthermore, the participants shared that participating in activities arranged by third sector organizations benefitted their mental health. However, it should be acknowledged that this result can partly be explained by the fact that the participants were recruited via these organizations, so each participant was familiar and already involved with these kinds of activities. Nevertheless, it can be argued that organizations like these create a safe space for immigrants, enable them to be involved in the community and openly communicate with others in a trustworthy environment. This corresponds well with the findings of Robertson et al. (2018), namely, promoting the mental health of males strengthens the social integration process by decreasing isolation.

In addition to meaningful activities, engaging with family, friends, and the whole community was mentioned by almost all participants in relation to their mental health. It is well established that maintaining close relationships with friends and family is beneficial to mental health (Fusar‐Poli et al., 2020), yet the immigrants seemed to highlight the support from their network as very important (Renner et al., 2020). Participants described this as comprehensive support including social, psychological, and practical dimensions that resulted in feelings of empowerment. Relationships with friends and family have also been suggested to facilitate professional help‐seeking among immigrants (McCann et al., 2016). Overall, the finding indicates that immigrants and the native population have similar perceptions of which factors promote their mental well‐being, yet show stark differences in the utilization of mental health services (e.g., Derr, 2016; Kieseppä et al., 2020). This suggest that family and community are important to mental health promotion among immigrant populations, and that the close bonds enable the provision of certain unofficial services for mental health problems. Nevertheless, the aspect of family and community can also negatively impact mental health among immigrant populations, via honor dynamics and other forms of domestic violence.

Concerning mental health treatment, immigrants preferred unprofessional help over professional services, which corroborates previous findings (Bettman et al., 2015; Mölsä et al., 2017; Renner et al., 2020; Satinsky et al., 2019; Valibhoy et al., 2017). The participants also mentioned several cultural barriers toward help‐seeking. Nevertheless, a majority implied that they would seek professional help if they felt the need. At the same time, most of the immigrants' comments suggested that they did not consider mental health problems, such as depressive symptoms, worthy of professional help. This indicates, at least to some degree, a lack of knowledge of mental health problems, which could be ameliorated by improving mental health literacy (Gele et al., 2016). Furthermore, the immigrants’ comments also imply that the mental health issue has potentially continued for an extended period of time, and reached a severe level, by the time professional help is requested. This can significantly prolong the recovery process. In relation to severe mental health illnesses, participants were in agreement that these required professional services. When asked about structural barriers to help‐seeking, immigrants mentioned a lack of knowledge regarding services and language difficulties, which agree with what has been reported in previous studies (e.g., Doğan et al., 2019; Kiselev et al., 2020; Renner et al., 2020; Satinsky et al., 2019; Valibhoy et al., 2017).

The participants of this study did not readily seek professional mental health services, but rather turned to peer support from their community. This possible barrier to help‐seeking can be removed by developing culturally sensitive mental health services that immigrants are comfortable using. This type of development would require experts with an immigrant background who can provide reliable insight into which aspects of care the immigrants are most uncomfortable with. Moreover, the provision of community‐based services that involve people with immigrant backgrounds could make mental health services more acceptable to immigrants from different cultures.

The participants also shared their ideas for how the utilization rates of mental health services among immigrants could be improved. The immigrants stressed that these services should be more flexible and include a low threshold for seeking help. In addition, they emphasized that recruiting staff from the immigrant population could improve access to mental health services. These types of interventions, that is, ensuring that people with immigrant backgrounds are employed in public services, have already shown promising results (WHO Europe, 2018a, 2018b). The participants also demonstrated a need for diverse information among immigrants, with some respondents suggesting that they would require information outside of the mental health sphere.

### 6.1 Strengths and limitations

The presented results describe immigrant perceptions of mental health promotion and treatment and provide unique insight for how Finnish health care can be developed in the future. The participants showed a high degree of heterogeneity and, as such, are a fair representation of the immigrant population. The results of the analysis were interpreted by two authors (N. G. and E. K.) to increase the trustworthiness of the results.

The difficulty in obtaining a representative sample of immigrant population underscores the difficulty of implementing this type of study. Collaboration with third sector organizations designated to increase our probability of getting participants for this study. Most of participants in this study participated via organization which operates only with women, and this affected participants gender distribution. Reason for immigration was not exclusion criteria which affected varying in participants' background.

However, as participation was voluntary, it is possible that the presented findings reflect the opinions of immigrants who are more competent and comfortable to talk and discuss issues regarding mental health. Another limitation was the fact that only partial data were collected via individual interviews, with some data coming from joint interviews, which means that data collection was not highly structured. This approach, however, increased participation in the study. This study applied semistructured interviews and thematic analysis to obtain qualitative information on immigrants' perceptions of the factors that support their mental health and mental health care in Finland. This approach was used to gain insight into individuals’ perceptions of this sensitive phenomenon. The sample size was small, but qualitative research does not necessarily require large sample sizes as there were clear signs of data saturation after the conducted interviews.

### 6.2 Implications

These findings can be utilized for planning and creating culturally sensitive mental health services. Contemporary decision‐making should always acknowledge cultural sensitivity when developing mental health services. Addressing the distinct needs of immigrants might improve the rate at which immigrants utilize mental health services. For example, mental health services should invest in using adequately trained interpreters and also seek to recruit people with immigrant backgrounds to both provide care and develop the provided services. Health care staff working with immigrants need proper education and knowledge to provide sufficient, holistic care; for this reason, information about the cultural differences among immigrants and cultural sensitivity should be included in basic as well as post‐graduate nursing education. Further research is needed to better understand how the utilization rates of mental health services can be improved within immigrant populations.

## 7 CONCLUSIONS

Immigrants use self‐help strategies to maintain their mental health at similar rates as the rest of the Finnish population. However, immigrants tend to seek professional help only once their mental health problems have reached a severe point; this shows that culturally sensitive mental health services need to be developed to meet the diverse needs of the immigrant population.

## CONFLICTS OF INTEREST

The authors declare no conflicts of intrest.

## Data Availability

The data that support the findings of this study are available on request from the corresponding author. The data are not publicly available due to privacy or ethical restrictions.
